# Elemental Sulfur Formation by *Sulfuricurvum kujiense* Is Mediated by Extracellular Organic Compounds

**DOI:** 10.3389/fmicb.2019.02710

**Published:** 2019-11-27

**Authors:** Brandi Cron, Pauline Henri, Clara S. Chan, Jennifer L. Macalady, Julie Cosmidis

**Affiliations:** ^1^Department of Geosciences, The Pennsylvania State University, University Park, PA, United States; ^2^Department of Earth Sciences, University of Delaware, Newark, DE, United States

**Keywords:** elemental sulfur, organomineralization, biomineralization, microbe-mineral interactions, organic-mineral interactions

## Abstract

Elemental sulfur [S(0)] is a central and ecologically important intermediate in the sulfur cycle, which can be used by a wide diversity of microorganisms that gain energy from its oxidation, reduction, or disproportionation. S(0) is formed by oxidation of reduced sulfur species, which can be chemically or microbially mediated. A variety of sulfur-oxidizing bacteria can biomineralize S(0), either intracellularly or extracellularly. The details and mechanisms of extracellular S(0) formation by bacteria have been in particular understudied so far. An important question in this respect is how extracellular S(0) minerals can be formed and remain stable in the environment outside of their thermodynamic stability domain. It was recently discovered that S(0) minerals could be formed and stabilized by oxidizing sulfide in the presence of dissolved organic compounds, a process called S(0) organomineralization. S(0) particles formed through this mechanism possess specific signatures such as morphologies that differ from that of their inorganically precipitated counterparts, encapsulation within an organic envelope, and metastable crystal structures (presence of the monoclinic β- and γ-S_8_ allotropes). Here, we investigated S(0) formation by the chemolithoautotrophic sulfur-oxidizing and nitrate-reducing bacterium *Sulfuricurvum kujiense* (Epsilonproteobacteria). We performed a thorough characterization of the S(0) minerals produced extracellularly in cultures of this microorganism, and showed that they present all the specific signatures (morphology, association with organics, and crystal structures) of organomineralized S(0). Using “spent medium” experiments, we furthermore demonstrated that soluble extracellular compounds produced by *S. kujiense* are necessary to form and stabilize S(0) minerals outside of the cells. This study provides the first experimental evidence of the importance of organomineralization in microbial S(0) formation. The prevalence of organomineralization in extracellular S(0) precipitation by other sulfur bacteria remains to be investigated, and the biological role of this mechanism is still unclear. However, we propose that sulfur-oxidizing bacteria could use soluble organics to stabilize stores of bioavailable S(0) outside the cells.

## Introduction

Elemental sulfur [S(0)] is an intermediate in the biogeochemical sulfur cycle, which can be found in many different types of environments such as marine sediments (Jørgensen and Nelson, 2004; Zopfi et al., 2004) and water columns (Findlay et al., 2014), euxinic lakes (Zerkle et al., 2010), sulfidic caves (Hamilton et al., 2015), hydrothermal vents (Taylor et al., 1999), as well as cold (Gleeson et al., 2011) or hot springs (Kamyshny et al., 2014). Its central position in the sulfur cycle makes it an ecologically important intermediate, as it can be consumed by a wide diversity of microorganisms that can gain energy from its oxidation (Franz et al., 2007; Marnocha et al., 2016), reduction (Boyd and Druschel, 2013), or disproportionation (Canfield and Thamdrup, 1994; Finster et al., 1998).

In low-temperature environments, S(0) is formed by the oxidation of more reduced sulfur species such as sulfide. This oxidation can occur chemically in the presence of oxygen or oxidized metals [e.g., Fe(III); Rickard and Luther, 2007], or it can be biologically mediated by diverse chemotrophic and phototrophic sulfur-oxidizing bacteria (SOB) and archaea (Dahl and Friedrich, 2008). The chemical oxidation of sulfide to S(0) by molecular oxygen occurs at rates that are several orders of magnitude lower than measured rates of microbial sulfide oxidation (Luther et al., 2011), and so it is usually assumed that in low-temperature environments, S(0) formation mostly results from microbial oxidation. Many different bacteria and archaea are able to biomineralize S(0) in the form of intra- or extra-cellular S(0) globules (Kleinjan et al., 2003; Dahl and Prange, 2006) or extracellular S(0) filaments (Wirsen et al., 2002; Sievert et al., 2007).

The details and mechanisms of extracellular S(0) formation by SOB have been understudied compared with those of intracellular microbial S(0). For instance, the vast majority of studies focusing on determining the chemical form and structure of microbial sulfur have considered only intracellular S(0) globules (e.g., Pasteris et al., 2001; Pickering et al., 2001; George et al., 2008; Berg et al., 2014). However, extracellular S(0) is probably more important than intracellular S(0) in terms of quantitative environmental accumulation, simply because the formation of intracellular S(0) globules is physically constrained by the limits of the bacterial cell wall. Moreover, extracellular S(0) might play a more important ecological role than intracellular S(0), as the S(0) produced by one population can, in theory, be used as an energy source by other populations of microorganisms (e.g., Warthmann et al., 1992). Furthermore, extracellular S(0) is potentially more relevant than intracellular S(0) for biotechnological applications, as it can be more easily separated from the microbial cells (Kleinjan et al., 2003).

The mechanism of extracellular S(0) formation by SOB is somewhat enigmatic. The enzymes catalyzing the oxidation of sulfide or thiosulfate to S(0) are usually located in the microbial periplasm (Gregersen et al., 2011), but S(0) globules are observed to nucleate and grow outside of cells, and sometimes at distance from the cells (Marnocha et al., 2016). It has thus been proposed that reduced sulfur species could be first oxidized to soluble intermediates such as polysulfides that could be exported and form S(0) globules extracellularly. Polysulfides were detected in cultures of the phototropic SOB *Chlorobaculum tepidum* during extracellular S(0) formation (Marnocha et al., 2016). However, polysulfides can also form from the chemical reaction of sulfide with S(0) (Kleinjan et al., 2005). It is also not clear how, once formed, extracellular S(0) can accumulate in the environment. Indeed, elemental sulfur is only thermodynamically stable under a very restricted range of Eh (−0.05 to 1.5) and pH (<5) conditions (Rickard and Luther, 2007), suggesting that it can only be present in environments where it is continuously being produced and/or where stabilizing mechanisms exist.

Recently, a new process called S(0) organomineralization was described (Cosmidis et al., 2019), which sheds new light on the mechanism of S(0) formation and stabilization in the environment. In this process, stable S(0) particles are formed by chemical oxidation of sulfide in the presence of dissolved organics. Importantly, S(0) is produced through this mechanism even under physicochemical conditions where no stable S(0) particles would form in the absence of organics, demonstrating that S(0) formation and stabilization are strongly controlled by interaction of sulfur with organics in solution. Organomineralized S(0) possesses properties that differ from that of inorganically precipitated S(0). Indeed, organomineralized S(0) particles have unusual morphologies, such as filaments branching at 45 and 90° angles, twisted filaments, or other complex shapes [often found in mixture with micrometric S(0) spheres]. They are furthermore commonly encapsulated within an organic envelope, and they finally contain metastable crystalline forms of S(0) such as the monoclinic β- and γ-S_8_ allotropes (Cosmidis and Templeton, 2016; Cosmidis et al., 2019). S(0) organomineralization was observed in experiments under a wide range of geochemical conditions, including conditions that prevail in many Earth-surface sulfidic environments (Cosmidis et al., 2019), suggesting that organics could play a previously unexpected role in S(0) formation and stabilization in nature.

The potential control on S(0) formation and stabilization by organics in solution through organomineralization is currently not taken into account in models of extracellular S(0) formation by microorganisms. We hypothesized that SOB could be excreting organics that help form and stabilize S(0) outside of the cells *via* an organomineralization process. This could explain certain puzzling properties of microbial extracellular S(0), such as its ability to form at distance from the cells (Marnocha et al., 2016) or the fact that it can sometimes be coated by organics (Hanson et al., 2016; Marnocha et al., 2019). Here, we present results from experiments conducted to test this hypothesis, using the chemolithoautotrophic SOB *Sulfuricurvum kujiense.*

*S. kujiense* was grown anaerobically in a mineral (organic-free) medium in the presence of sulfide or thiosulfate as electron donors, and nitrate as an electron acceptor. Under these conditions, *S. kujiense* grew autotrophically and produced extracellular S(0) particles. Using multiple microscopy and spectromicroscopy methods, we characterized the extracellular S(0) biominerals produced by this organism, and compared their properties (morphology, association with organics, and crystal structure) with that of organomineralized S(0). We furthermore designed an experiment using the “spent medium” of *S. kujiense* to test the role of dissolved organics secreted by the bacteria in the extracellular medium on S(0) precipitation. This study is the first experimental investigation of the role of microbially produced organics on the formation and stabilization of extracellular S(0) minerals by SOB.

## Methods

### Experimental

#### *Sulfuricurvum kujiense* Cultures

*Sulfuricurvum kujiense* is a facultatively anaerobic, chemolithoautotrophic sulfur-oxidizing Epsilonproteobacterium (Kodama and Watanabe, 2004; Han et al., 2012). It can grow anaerobically using thiosulfate or sulfide as the electron donor and nitrate as the electron acceptor (Kodama and Watanabe, 2004). A *S. kujiense* culture was obtained from the Leibniz Institute DSMZ (German Collection of Microorganisms and Cell Cultures). Culture bottles containing DSMZ medium 1020 were sealed and sparged for 30 min to 1 h (depending on the volume) with a CO_2_/N_2_ (20/80) gas mixture. For some experiments, sodium sulfide (Na_2_S) was added as a redox buffer (20 μM). Culture bottles were sealed with rubber stoppers and the headspace was flushed for 15 s with H_2_ gas to 0.5 bar overpressure. After autoclaving, bicarbonate (0.2 M) was added to buffer the media to pH 7, and either filter-sterilized thiosulfate (Na_2_S_2_O_3_, 10 mM final concentration) or filter-sterilized sulfide (Na_2_S, 2 mM final concentration) and thiosulfate (Na_2_S_2_O_3_, 10 mM final concentration) were added. The sterile media were then inoculated with living *S. kujiense*. Uninoculated abiotic controls were also prepared. Particles formed in the experimental media were collected by centrifugation at different time points and prepared for analyses (see Supplementary Table S1 for a summary of experimental conditions and sampling times).

#### *Sulfuricurvum kujiense* Spent Medium Experiments

Spent medium experiments were designed to test the role of soluble extracellular compounds produced by *S. kujiense* in the formation or stabilization of S(0). For these experiments, two different cultures of *S. kujiense* in DSMZ medium 1020 were used. The first spent medium experiment was prepared with cultures allowed to grow for more than 1 month, corresponding to the stationary phase (no cell counts were made). Cells were grown from cultures supplemented with 10 mM final concentration of Na_2_S_2_O_3_ and 2 mM final concentration of Na_2_S. Another spent medium experiment was prepared with cultures supplemented with thiosulfate (10 mM) only and allowed to grow for 4 days, corresponding to the exponential phase (cell density ~3.6 × 10^4^ cells ml^−1^). Both types of cultures (stationary and exponential) were filtered on 0.2 μm filters to remove cells, and the sterile filtrates are hereafter referred to as “spent media.” A Shimadzu TOC-V_CPH_ Total Organic Carbon Analyzer was used to measure the amount of total dissolved organic carbon (DOC) in the spent media. The measured DOC concentrations in the spent media were 7 mg C L^−1^ for the exponential phase medium, and 60 mg C L^−1^ for the stationary phase medium. The pH of the spent media was ~8 at time of collection, and adjusted to ~6.5 using hydrochloric acid (1 M). pH adjustments were necessary to keep the pH of the spent medium experiments within the pH range of *S. kujiense* cultures after subsequent sodium sulfide addition. Sterile bottles containing 500 ml volumes of the spent media were sparged with a CO_2_/N_2_ (20/80) gas mixture for 15 min in a biosafety hood. A filter-sterilized Na_2_S solution was immediately added to a final concentration of 500 μM, and the bottles were loosely capped in order to let oxygen from the atmosphere diffuse in. No thiosulfate was added since thiosulfate is not known to oxidize to S(0) at significant rates in the absence of microbial activity (Houghton et al., 2016). An abiotic control, consisting of an uninoculated DSMZ 1020 medium with Na_2_S_2_O_3_ (10 mM), filter-sterilized, sparged with CO_2_/N_2_ (20/80), and supplemented with 500 μM Na_2_S, was also prepared and allowed to oxygenate under the same incubation conditions. The experiments were kept in the dark at room temperature, and samples were collected at different times for analyses (see Supplementary Table S2). The pH of the experiments and abiotic control was ~8.5, 1 day after sulfide addition, and remained constant until the end of the study (~6 months later).

Note that the spent medium experiments were conducted in the presence of oxygen, and are thus not testing the potential for organics to oxidize sulfide to S(0) (sulfide was chemically oxidized by O_2_). These experiments were rather testing the potential for extracellular organics to stabilize oxidized sulfur as S(0) particles.

#### *Escherichia coli* Spent Medium Experiment

An *Escherichia coli* spent medium experiment was prepared in order to compare the S(0)-forming properties of soluble extracellular compounds produced by *S. kujiense* with those of a non-sulfur-oxidizing microorganism. *E. coli* (strain MOD1-EC6074 from the Penn State *E. coli* reference center) was grown in LB at 37°C. After 48 h, cells were harvested by centrifugation, rinsed six times with deionized water, and transferred to DSMZ medium 1020 at a density of ~10^13^ cells L^−1^. Thiosulfate (10 mM) and bicarbonate (20 mM) were then added to mimic *S. kujiense* culture conditions. After 7 days, the culture medium was filtered on 0.2 μm filters to remove *E. coli* cells, and its pH was adjusted to 6.5 using hydrochloric acid (1 M). A 500 ml volume was sparged with a CO_2_/N_2_ (20/80) gas mixture for 15 min, and filter-sterilized Na_2_S was immediately added under sterile conditions at a 500 μM final concentration, similar to the S*. kujiense* spent medium experiments. The initial DOC concentration of the *E. coli* spent medium was 12 mg C L^−1^. The experimental bottle was loosely capped and kept in the dark at room temperature until sample collection at 2 months for S K-edge XANES analyses, and at 5 months for SEM. The pH was ~8.5, 1 day after sulfide addition, and remained constant until the end of the study (5 months later).

### Analytical

#### Scanning Electron Microscopy

For Scanning Electron Microscopy (SEM) analyses, *S. kujiense* cultures and spent medium experiments were filtered onto polycarbonate filters (GTTP Isopore membrane filters, Merck Millipore, pore size 0.2 μm), and rinsed with deionized (DI) water. For correlative SEM and Raman spectromicroscopy, a sample from a *S. kujiense* spent medium experiment was centrifuged and the pellet was rinsed three times with DI water to remove salts. A 20 μl volume was then deposited and dried on a glass microscope slide. SEM analyses were conducted on a Field Emission Nova NanoSEM 630 scanning electron microscope at the Materials Characterization Laboratory of the Pennsylvania State University. Images were acquired in the secondary electron mode and the backscattered electron mode with the microscope operating at 3–7 kV and a working distance (WD) of ~3–5 mm. Energy-Dispersive X-ray Spectrometry (XEDS) analyses were performed at 12 kV and WD ~7 mm.

#### Scanning Transmission X-ray Microscopy

For Scanning Transmission X-ray Microscopy (STXM) analyses, 5 ml of a *S. kujiense* culture was pelleted and rinsed three times with DI water to remove salts. About 1 μl volumes of the resuspended pellet were deposited on Formvar-coated 200 mesh Cu TEM grids (Ted Pella) and allowed to air-dry. STXM analyses were performed on beamline 10ID-1 (SM) of the Canadian Light Source (Saskatoon, Canada) (Kaznatcheev et al., 2007). After sample insertion, the STXM chamber was evacuated to 100 mTorr and back-filled with He at ~1 atm pressure. The X-ray beam was focused on the samples using a 35 nm Fresnel zone plate objective and an order-sorting aperture, yielding a focused X-ray beam spot of ~40 nm. Energy calibration was achieved using the well-resolved 3p Rydberg peak of gaseous CO_2_ at 294.96 eV. Images, maps, and image stacks were acquired in the 260–340 eV (C K-edge) and 155–190 eV (S L-edge) energy ranges. The aXis2000 program^1^ was used for data processing. A linear background correction was applied to the spectra at the C K-edge (in the 260–280 eV region) and S L-edge (in the 155–160 eV region), to eliminate contributions of lower energy absorption edges.

Maps of organic carbon were obtained by subtracting an image obtained at 280 eV (i.e., below the carbon K-edge) and converted into optical density (OD) from an OD-converted image at 288.2 eV (1 s → π* electronic transitions of C in amide groups). Maps of S were obtained by subtracting an OD-converted image obtained at 160 eV (i.e., below the S L-edge) from an OD-converted image at 163.5 eV (energy of the S L_3_-edge). X-ray Absorption Near-Edge Structure (XANES) spectra were extracted from image stacks as detailed in Cosmidis and Benzerara (2014). S L-edge XANES spectra were compared to that of a reference elemental sulfur compound (precipitated sulfur, Alfa Aesar).

#### Fourier-Transform Infrared Spectroscopy

For Fourier-Transform Infrared Spectroscopy, 5 ml volumes of the *S. kujiense* spent medium experiment and of the abiotic control were pelleted and rinsed three times with DI water, and concentrated suspensions were deposited and air-dried on aluminum foils. FT-IR measurements were conducted on a vertex 70 infrared spectrometer (Bruker Optics) equipped with a deuterated triglycerine sulfate (DTGS) detector and high intensity water cooled globar source. Spectra were collected at 5 cm^−1^ resolution (2.5 mm aperture) as an average of 100 scans using MVP PRO (Harrick Scientific) diamond attenuated total reflectance (ATR) accessory set at a fixed incident angle of 45°. The instrument was purged for 30 min before the first measurement to ensure baseline stability. Data were baseline corrected using the “Rubber Band” algorithm within the OPUS 2.2 software. The experimental spectra were plotted with a hydroxyapatite reference spectrum (RRUFF database; Lafuente et al., 2015). Except when otherwise noted, tentative band and peak assignments were performed according to Bellamy (1975) and Socrates (2004).

#### Raman Spectromicroscopy

Raman spectromicroscopy was used to investigate the crystal structure of S(0) particles formed in *S. kujiense* cultures and spent medium experiments. About 5 ml samples were concentrated by centrifugation and rinsed three times with DI water. The *S. kujiense* culture sample was deposited on a microscope glass slide and kept wet during analyses. The sample from the *S. kujiense* spent medium experiment was dried on a microscope slide for correlative Raman and SEM analyses. Raman spectra were collected using a Horiba LabRam HR Evolution Raman Vis-NIR spectrometer coupled with a HeNe 633nm laser source. Spectra were collected using a Si-based CCD detector, and processed using the LabSpec 6 software (Horiba Scientific). The spectrometer was calibrated using the 520 cm^−1^ Raman peak of Si prior to analysis. Spectral data were corrected for instrumental artifacts and baseline-subtracted using a polynomial fitting algorithm in LabSpec 6. Ultra-Low Frequency (ULF) Raman measurements were collected in the 10–100 cm^−1^ range as described in Nims et al. (2019). Experimental spectra were compared with reference spectra for the elemental sulfur allotropes α-, β-, and γ-S_8_ (Nims et al., 2019).

#### X-ray Absorption Near-Edge Structure Spectroscopy at the S K-Edge

X-ray Absorption Near-Edge Structure (XANES) spectroscopy at the S K-edged was used to investigate sulfur speciation in solution in *S. kujiense* cultures and in the spent medium experiments. The following solutions were collected: (1) a “blank” solution corresponding to DSMZ medium 1020 with thiosulfate; (2) a solution from a culture of *S. kujiense* in DSMZ medium 1020 with thiosulfate at 4 days; (3) a solution from the stationary phase *S. kujiense* spent medium experiment after sulfide addition and oxygenation for 6 months; (4) a solution from a *E. coli* spent medium experiment after sulfide addition and oxygenation for 2 months. In every case, ~5 ml of the solution was collected and filtered on a 0.2 μm polycarbonate filter to remove particulate materials, and immediately frozen at −20°C. The samples were kept frozen during transport to the beamline. XANES analyses were performed on beamline 4–3 of the Stanford Synchrotron Radiation Lightsource (SSRL, Standford, USA). At the beamline, each solution sample was quickly thawed and injected into a sample holder where it was contained between sulfur-free XRF tape (Premier Lab Supply) and sulfur-free Mylar X-ray film (Premier Lab Supply). The sample was then immediately frozen in place using a liquid He cryostat, which maintained the temperature at ~25 K during XANES measurements. Analyses were performed in fluorescence-yield mode using a Stern-Heald ionization detector. The Si(111) double crystal monochromator was calibrated using a thiosulfate standard, by setting the position of the maximum of the first pre-edge feature to an energy of 2472.0 eV.

Up to five scans per sample were recorded and averaged to improve the signal-to-noise ratio. A linear background determined in the pre-edge region (2400–2,470 eV) was subtracted from the averaged data, and the spectra were then normalized at 2510 eV using the Athena program (Ravel and Newville, 2005). Gaussian curve fitting was performed in the 2,465–2,490 eV energy range using Athena, as described for instance in Manceau and Nagy (2012). For the blank sample as well as for the *S. kujiense* culture and the *E. coli* spent medium experiment, the initial energy of the first arctangent function was set below the 2,476 eV and the initial energy of the second arctangent was set above the energy of 2482.75 eV. No constraints were placed on the widths of the arctangents or the Gaussian curves. The quality of the fits was evaluated based on *χ*^2^ values and *R* factors.

The experimental spectra were compared with reference spectra from solid sulfur-bearing standards on sulfur-free XRF tape, acquired and processed under the same conditions: cystine, sodium thiosulfate, magnesium sulfate, elemental sulfur, and polysulfides ($S_{n}^{2-}$; *n* = 5).

## Results

### Characterization of Extracellular S(0) Particles in *Sulfuricurvum kujiense* Cultures

**Figure 1:**
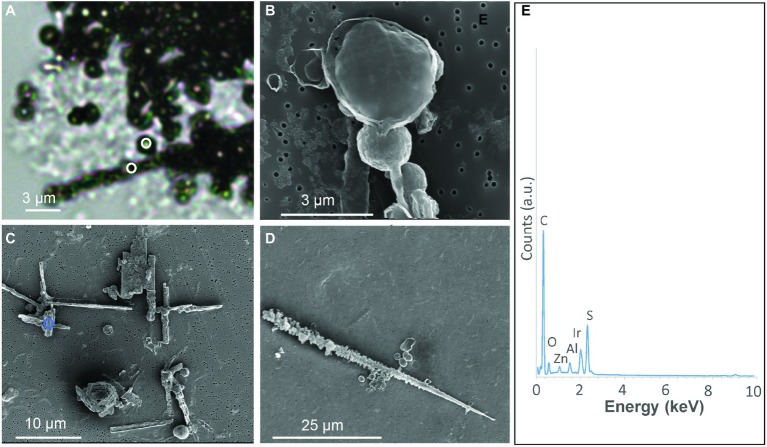
S(0) particles formed in *S. kujiense* cultures with sulfide and thiosulfate. **(A)** Light micrograph of sulfur spheres and rods observed after 2 weeks of growth. White circles in **(A)** indicate where Raman spectra shown in Figure 2 were collected. **(B)** SEM image of spheres observed at 4 days. **(C–D)** SEM images of spheres and rods observed at 2 weeks. **(E)** XEDS spectrum collected on the area depicted by a blue circle in **(C)**. Iridium (Ir) originates from the conductive coating of the SEM sample, while zinc (Zn) and aluminium (Al) are thought to originate from the sample holder and instrument.

*S. kujiense* was grown anaerobically in a mineral medium using either thiosulfate and sulfide or thiosulfate only as electron donors, and nitrate as the electron acceptor. Oxidation of reduced sulfur species by *S. kujiense* lead to the precipitation of S(0) particles after a few days when both thiosulfate and sulfide were provided (Figure 1; Supplementary Figure S1). However, no S(0) was not formed in the *S. kujiense* culture supplemented with thiosulfate only after 5 weeks, and only Ca,Mg-phosphate particles were found (Supplementary Figure S2). S(0) was also absent from the uninoculated abiotic controls with both sulfide and thiosulfate at any time point.

**Figure 2:**
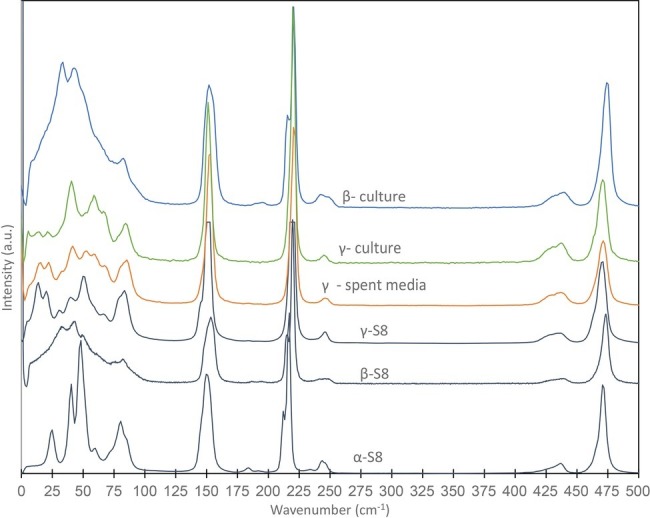
Raman spectra obtained on S(0) particles formed in a *S. kujiense* culture with thiosulfate and sulfide, and in a *S. kujiense* spent medium experiment. The spectra were acquired on particles shown in Figure 1A (“β-culture” and “γ-culture”) and Figures 4D–I (“γ-spent media”). Spectra for α-S_8_, β-S_8_, and γ-S_8_ standards are also shown. Differences in peak intensities between the samples and the reference spectra are due to crystal orientation effects.

**Figure 3:**
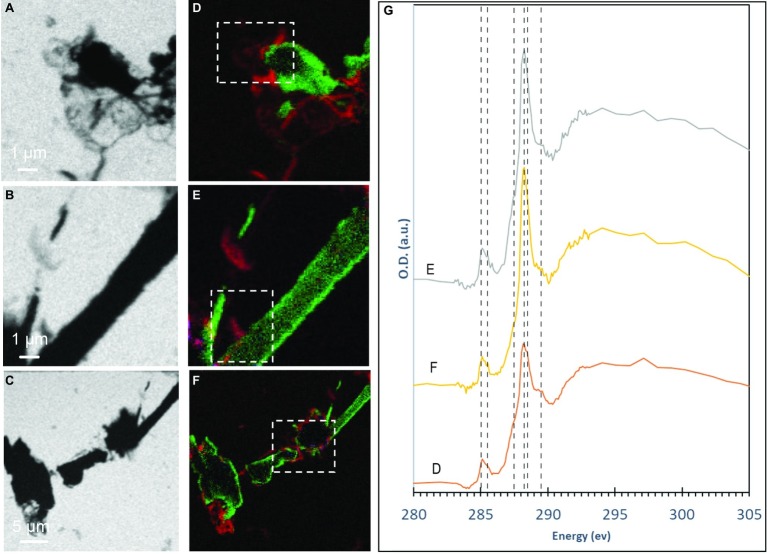
STXM analyses of S(0) particles produced by *S. kujiense* in cultures with sulfide and thiosulfate after 5 weeks. **(A–C)** STXM images collected at carbon K-edge (288.2 eV). **(D–F)** map overlays: green areas are rich in sulfur and red areas are rich in organic carbon. **(G)** XANES spectra at the carbon K-edge obtained on the particles shown in panels **(D–F)** (dashed boxes). Vertical lines in **(G)** are located at 285 eV and 285.5 eV (1 s → π* transitions in aromatics), 287.44 eV (3 s → σ* transitions in aliphatics), 288.2 eV (1 s → π* transitions in amide groups of proteins), 288.5 eV (1 s → π* transitions in carboxylic groups), and 289.5 eV (1 s → σ* transitions in alcohols).

The S(0) particles produced by *S. kujiense* in the presence of both thiosulfate and sulfide appeared in light micrographs and SEM images as spheres ~1–3 μm in diameter, rods 10–50 μm in length, sometimes with short branches (Figure 1D), and more irregular shapes (Figure 1C). Rods were frequently closely associated with or covered by spheres (Figures 1A,C). The presence of S(0) in these particles was confirmed by XEDS (Figure 1E), and STXM analyses at the S L-edge (Supplementary Figure S3). These S(0) particles produced by *S. kujiense* were identified as crystals of the monoclinic allotropes β- and γ-S_8_ using ULF Raman spectromicroscopy (Figure 2). STXM at the C K-edge furthermore revealed that these S(0) particles were covered by a thin envelope of organic carbon (Figure 3), which is probably responsible for the smooth aspect of the particles’ surfaces in SEM images (see for instance Figure 1B, where the organic envelope around a sphere is clearly visible). The organic envelope is also apparent in STXM images where S(0) has been sublimated away in the vacuum of the STXM chamber (see for instance the empty organic vesicles in Figure 3A). Carbon K-edge XANES spectra obtained on these organic envelopes (Figure 3G) present a main peak at 288.2 eV, corresponding to 1 s→π^*^_C=O_ electronic transitions in amide groups, and/or at 288.5 eV, corresponding to 1 s→π^*^_C=O_ electronic transitions in carboxylic groups, depending on the particles. Smaller peaks or shoulders are also present in all the collected spectra at 285.0 and 285.5 eV (1 s→π^*^_C=C_ transitions in aromatic or unsaturated carbon), 287.4 eV (3 s→σ* transitions in aliphatics), and 289.5 eV (1 s→σ* transitions in alcohols). Peak assignments were made according to Benzerara et al. (2004), Haberstroh et al. (2006), Lehmann et al. (2007, 2009), Solomon et al. (2009), and Moffet et al. (2011).

### Formation of Elemental Sulfur Minerals in the Spent Medium of *Sulfuricurvum kujiense*

“Spent medium” experiments were performed, where cultures of *S. kujiense* in DSMZ medium 1020 were filter-sterilized to remove cells, and sulfide was added as Na_2_S (500 μM). The cultures were either in the exponential growth phase or in the stationary phase at time of filtration. After sulfide addition, oxygen from the atmosphere was allowed to diffuse in the experimental bottles. Two controls were also performed: (1) an abiotic control (i.e., the same experiment using uninoculated DSMZ medium 1020), and (2) an *E. coli* “spent medium” control (i.e., the same experiment using *E. coli* instead of *S. kujiense*). The goal of these experiments was to evaluate differences in S(0) formation from sulfide oxidation in the presence and absence of soluble extracellular compounds excreted by *S. kujiense*, and in the presence of soluble extracellular compounds produced by a different, non S-oxidizing microorganism (*E. coli*).

No S(0) particles were formed in the abiotic control that did not contain any soluble microbially derived organic compound. Instead, only a poorly crystalline calcium-phosphate phase could be detected using SEM/XEDS and FT-IR (Supplementary Figure S4).

**Figure 4:**
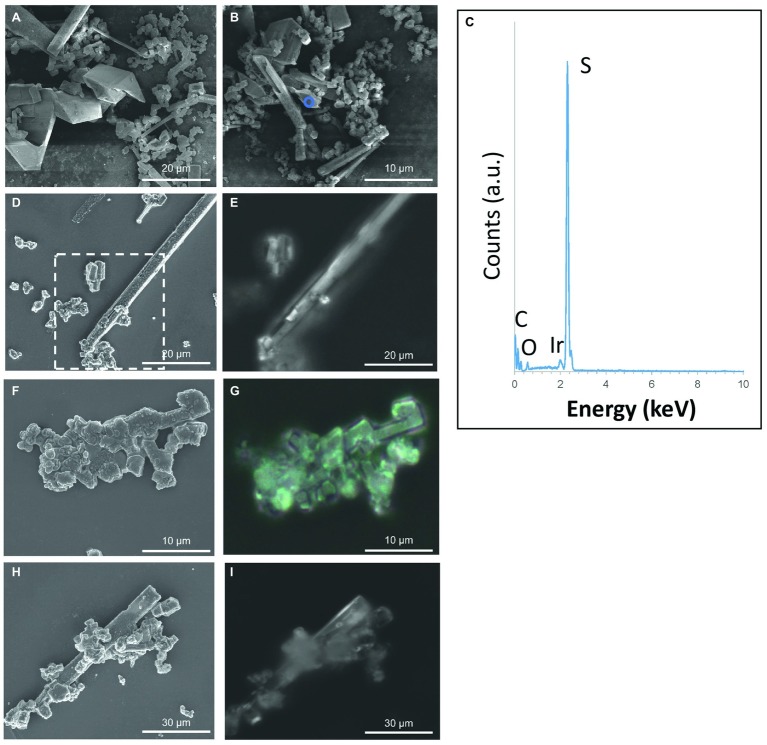
S(0) particles formed in the (stationary phase) *S. kujiense* spent medium experiment. **(A,B,D,F,H)** SEM images. **(C)** XEDS spectrum collected on the area depicted by the blue circle in **(B)**. **(E,G,H)** Correlative light microscopy images acquired with a Raman spectromicroscope on the areas corresponding to the white rectangle in image **(D)**, and to images **(F)** and **(H)**, respectively. Raman spectra were collected on the particles shown in **(D–I)** (see Figure 2). Note that an average of three Raman spectra were acquired on each particle, and that they were all similar to the representative spectrum shown in Figure 2.

**Figure 5:**
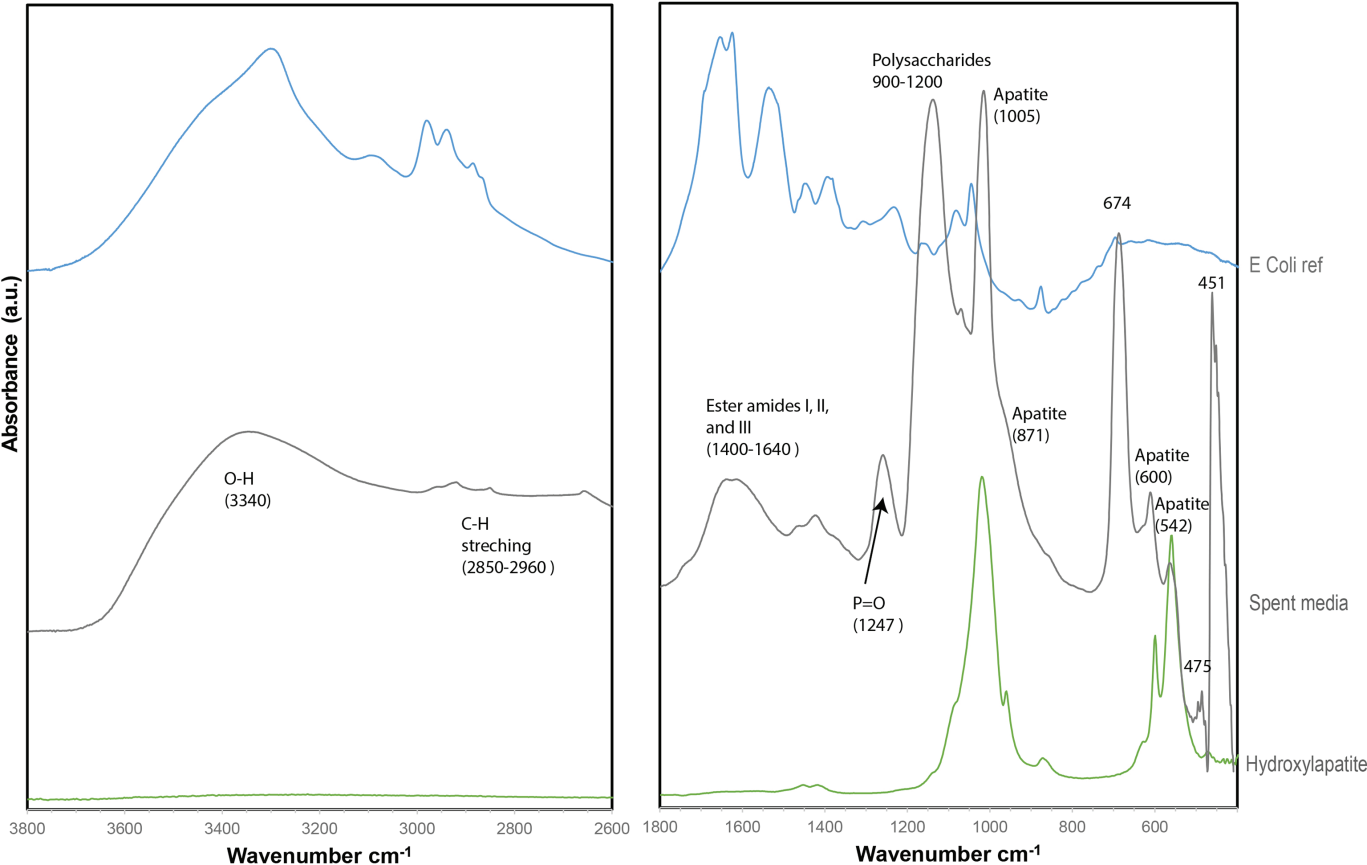
ATR-FTIR spectrum of particles from the (stationary phase) *S. kujiense* spent medium experiment (gray), plotted along with a reference *E. coli* spectrum (blue) and a hydroxylapatite reference spectrum (RRUFF database) (green).

No S(0) particles were formed after 5 weeks in the *S. kujuense* spent medium experiment prepared using the extracellular medium of cultures in the exponential phase. SEM-XEDS analyses revealed the presence of Ca, Mg, and P-rich particles (Supplementary Figure S5). In contrast, abundant S(0) minerals precipitated in the *S. kujiense* spent medium experiment prepared using cultures in the stationary phase (Figure 4). This difference might be explained by the fact that the spent medium from the stationary phase culture contained about 10 times more organics than the spent medium from the exponential phase culture (see Methods Section “***Sulfuricurvum kujiense*** Spent Medium Experiments”). The S(0) particles appeared as spheres (1–3 μm in diameter), elongated rods (10–20 μm in length) as well as prisms of different shapes and sizes (10–20 μm) that sometimes showed hollow interiors (Figure 4). These different types of particles were all composed of γ-S_8_, as shown by correlative SEM and ULF Raman spectromicroscopy (Figure 2). ATR-FTIR spectroscopy was used to detect and identify potential organics associated with these S(0) particles (Figure 5). Their ATR-FTIR spectra presented several peaks and bands that can be attributed to organic groups. A broad band centered around 3,400–3,430 cm^−1^ is attributed to O—H bond stretching frequencies, while peaks around 2,850–2,960 cm^−1^ correspond to C—H stretching frequencies. Broad bands around 1,400–1,640 cm^−1^ are tentatively attributed to ester amides I, II, and III. The broad band centered around 1,640 cm^−1^ might also be attributed to C=C stretching vibrations. The broad bands centered around 1,155 and 1,250 cm^−1^ might correspond to C—O—C and C—O—H stretching vibrations, whereas the small peak at 1060 cm^−1^ might correspond to C—O stretching frequencies in alcohols, aliphatic ethers of polysaccharides, or similar substances. The bands and peaks at 1155 and 1,060 cm^−1^ might be due to the presence of sulfurized organics (C=S stretch, and C-S stretch or S = 0 stretch in sulfoxides, respectively). Peaks at 475 and 451 cm^−1^ are attributed to S-S stretching in S_8_ (Meyer, 1976; Eckert and Steudel, 2003). Peaks, at 1005, 871, 600, and 542 cm^−1^, and a shoulder at ~950–871 cm^−1^ are attributed to phosphate minerals that co-precipitated with S(0).

In the *E. coli* spent medium experiment, both fine (~100 nm) Ca-Mg-phosphates and large (~10 μm) S-bearing particles most probably corresponding to S(0), were detected using SEM/XEDS (Supplementary Figure S6). The spent medium from *E. coli* contained ~12 mg C L^−1^, which is only slightly more DOC than the spent medium from the exponential phase *S. kujiense* culture (~7 mg C L^−1^), where no S(0) was found. This suggests that extracellular organics produced by *S. kujiense* are not particularly potent for S(0) formation and/or stabilization.

### Sulfur Speciation in Solution in Spent Medium Experiments

**Figure 6:**
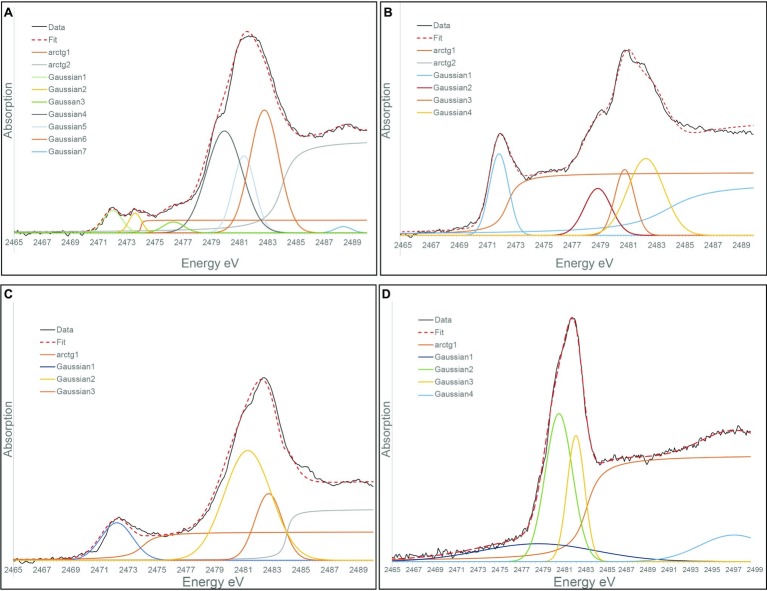
Gaussian curve fitting of S K-edge XANES spectra from: **(A)** A “Blank” solution corresponding DSMZ medium 1020 supplemented with thiosulfate. **(B)** The solution from 4-day-old *S. kujiense* culture in DSMZ medium 1020 with thiosulfate. **(C)** The solution from the *E. coli* spent medium experiment, 2 months after sulfide addition. **(D)** The solution from the stationary phase S*. kujiense* spent medium experiment 6 months after sulfide addition.

XANES at S K-edge was used to characterize the speciation of sulfur in solution in *S. kujiense* cultures and in spent medium experiments. The “blank” spectrum in Figure 6A corresponds to DSMZ medium 1020 supplemented with Na_2_S_2_O_3_. It could be fitted with Gaussian functions at 2472.0, 2479.9, and 2481.3 eV, which corresponds, with minor shifts (±0.01–0.6 eV), to the main peaks (2472.0, 2479.2, and 2480.8 eV) in the spectrum of a solid Na_2_S_2_O_3_ standard (Supplementary Figure S7D, Supplementary Table S3). It has to be noted that peak shifts are expected when comparing S K-edge XANES spectra from sulfur species in aqueous solutions versus solids (Pin et al., 2013).

Figure 6B shows the S K-edge XANES spectrum of a 4-day-old *S. kujiense* culture in to DSMZ medium 1020 supplemented with Na_2_S_2_O_3_. Peaks are present at 2472.0, 2480.9, 2482.4, and 2482.75 eV, which could be attributed to thiosulfate as well as sulfate (by comparison with a solid magnesium sulfate standard with a main peak at 2482.6 eV; Supplementary Figure S7A). The first peak at 2472.0 eV is however relatively taller compared with the “blank” spectrum, which might be due to the additional presence of a zero-valent sulfur component, such as S(0) in colloidal form, or an organic di- or poly-sulfide (see for instance the cystine reference spectrum with a main peak at 2472.5 eV; Supplementary Figure S7C). This peak might also be explained by the presence of long chain inorganic polysulfides (e.g., S_8_^2−^). Short chain inorganic polysulfides are excluded as their external sulfur atoms would generate an additional peak around 2,471 eV (Supplementary Figure S7E; Pascal et al., 2014).

The peaks in the spectrum from the solution of the *E. coli* spent medium experiment (2472.2, 2481.3, and 2482.75 eV) are consistent with the presence of thiosulfate plus sulfate (Figure 6C). The S K-edge XANES spectrum from the solution of the (stationary phase) *S. kujiense* spent medium experiment (Figure 6D) could be fitted with two main Gaussians at 2481.3 and 2482.75 eV, which could be interpreted as protonated (HSO_4_^−^) and deprotonated (SO_4_^2−^) sulfate ions in solution (Pin et al., 2013).

## Discussion

### Extracellular Sulfur Particles Formed by *Sulfuricurvum kujiense* Share Characteristics With Organomineralized S(0)

In this study, we characterized the S(0) particles formed in cultures of the sulfur-oxidizing chemolithoautotrophic bacterium *S. kujiense*. Below we compare the properties of these particles to that of organomineralized S(0) particles, i.e., S(0) particles formed abiotically by oxidation of sulfide in the presence of oxygen and dissolved organics (Cosmidis et al., 2019). We also compare them with different examples of biomineralized S(0) and inorganically precipitated S(0) from the literature.

The S(0) particles formed by *S. kujiense* are micrometric spheres and elongated rods (Figure 1). Elemental sulfur spheres are very common in S(0) organomineralization processes, and are always present in mixture with more complex morphologies (Cosmidis and Templeton, 2016; Cosmidis et al., 2019). Spherical S(0) is also a typical morphology for both intra- and extracellular microbial sulfur (Dahl and Prange, 2006), as well as for colloidal S(0) particles called sols (Steudel, 2003). Micrometer-sized S(0) spheres are thought to result from the crystallization of liquid-like sulfur droplets, the stability of which is increased in the presence of organic surfactants (Steudel, 1996, 2003). However, it is not clear whether spheres can be used as signatures of S(0) precipitated in the presence of organics, since they have also been described for inorganically-precipitated S(0) (see for instance the abiotic sols in Marnocha et al., 2019).

The rods can be compared to the rectilinear S(0) filaments formed in organomineralization experiments (see for instance Figures 2E,F in Cosmidis et al., 2019). Elongated S(0) particles such as needles are also typical of inorganically-precipitated monoclinic sulfur (Steudel and Eckert, 2003), so S(0) particles with rod shapes are not diagnostic for organomineralization processes. However, long rods with short radiating branches (Figures 1C,D) are very similar to the “test tube brush” morphologies described for organomineralized S(0) in Cosmidis et al. (2019), and have to our knowledge not been described for S(0) precipitated in the absence of organics.

STXM analyses of the S(0) particles precipitated by *S. kujiense* show that they are encapsulated within an organic envelope (Figure 3), which contains carboxylic groups, aliphatics, alcohols, and sometimes amide groups. This is very similar to S(0) particles formed in organomineralization experiments, which are always encapsulated within an organic envelope (Cosmidis et al., 2019). Interestingly, carboxylics are always the dominant functional group in the C K-edge XANES spectra of the organic envelopes of organomineralized S(0) particles, regardless of the type of organic compound used for organomineralization (Cosmidis et al., 2019), as is also the case in the XANES spectra of the particles formed by *S. kujiense*. Amide groups, which were present in the envelopes of some of the S(0) particles precipitated by *S. kujiense* (Figure 3G), have also been detected in the organic envelope of organomineralized S(0) when proteins were present in their experimental formation medium (for instance when yeast extract or peptone are used for organomineralization; Cosmidis and Templeton, 2016).

Finally, extracellular S(0) particles formed in cultures of *S. kujiense* correspond to the monoclinic sulfur allotropes β- and γ-S_8_ (Figure 2). β-S_8_ is thermodynamically unstable at temperatures below 96°C, while γ-S_8_ is metastable at all temperatures at ambient pressures (Steudel and Eckert, 2003). These two phases are unexpected but commonly observed in S(0) organomineralization experiments (Cosmidis et al., 2019). Based on several laboratory and field-based studies, the formation of β-S_8_ under low-temperature conditions is typical of sulfur precipitation in the presence of organics (Guo et al., 2006; Choudhury et al., 2013; Moon et al., 2013; Lau et al., 2017). γ-S_8_ can be obtained inorganically by quenching and stretching liquid sulfur (Steudel and Eckert, 2003), but in low-temperature environments γ-S_8_ is often found in close association with organics (Douglas and Yang, 2002; Gleeson et al., 2012; Lau et al., 2017).

The properties (morphology, crystal structure, and association with organics) of the extracellular S(0) particles formed in cultures of *S. kujiense* cultures are thus similar to that of organomineralized S(0). This suggests that organics likely play an important role in the formation and stabilization of these particles. *S. kujiense* cells were grown autotrophically in an organic-free medium (DSMZ medium 1,020). These organics were thus derived from the cells themselves. In the next section, we discuss the results of “spent medium” experiments, where we tested the effect of soluble extracellular compounds produced by *S. kujiense* to form and stabilize S(0) particles during chemical sulfide oxidation by oxygen.

### The Role of Extracellular Compounds Produced by *Sulfuricurvum kujiense* in the Formation and Stabilization of S(0) Particles

We observed the formation of S(0) particles in the spent medium of a stationary phase *S. kujiense* culture in which sodium sulfide was added and oxygen was allowed to diffuse. In the same medium (DSMZ medium 1020) where cells had not been growing (i.e., in the abiotic control), S(0) particles were not observed after sulfide addition and oxygenation. This demonstrates that soluble compounds produced in the extracellular medium during *S. kujiense* growth play an important role in the formation and stabilization of S(0) particles by this organism. We hypothesize that these soluble compounds are organics. Further investigation will be needed to identify the specific extracellular organic molecules produced by *S. kujiense* and involved in S(0) formation, using for instance hydrophobic interaction chromatography (e.g., Ni et al., 2015) or metabolomics approaches.

As described in the previous section, the S(0) particles produced by *S. kujiense* indeed possess properties similar to that of organomineralized S(0) [see Section “Extracellular Sulfur Particles Formed by *Sulfuricurvum kujiense* Share Characteristics With Organomineralized S(0)”]. These properties: (1) spherical morphologies, (2) association with organics, and (3) presence of monoclinic sulfur allotropes, were also reproduced in the S(0) products of the *S. kujiense* spent medium experiment, as shown by correlative SEM-Raman spectromicroscopy (Figure 4) and FT-IR analyses (Figure 5). The effect of organics on S(0) formation appears to be concentration dependent, as no S(0) was formed in the *S. kujiense* spent medium experiment from an exponential phase culture, which contained only 7 mg C L^−1^ (compared with 60 mg C L^−1^ for the stationary phase *S. kujiense* spent medium experiment). It is furthermore possible that different chemical compositions of the released organic material in the stationary versus exponential phase could affect the extent of S(0) production in *S. kuijense* spent medium experiments.

S(0) particles were also formed in the of *E. coli* spent medium experiment (Supplementary Figure S6). This spent medium contained ~12 mgl^−1^ DOC, confirming the importance of extracellular organics in the formation and stabilization of particulate S(0). The results of this control experiment with a non-sulfur-oxidizing bacterium suggest that a broad diversity of microbial species could produce extracellular organics that favorize extracellular S(0) formation. However, the S(0) particles found in the spent medium of *E. coli* have sizes and shapes that differ from those present in *S. kujiense* cultures or spent medium, indicating that the morphology of S(0) particles is controlled by the type of organics present during their formation, confirming previous results on organomineralized S(0) by Cosmidis et al. (2019).

**Figure 7:**
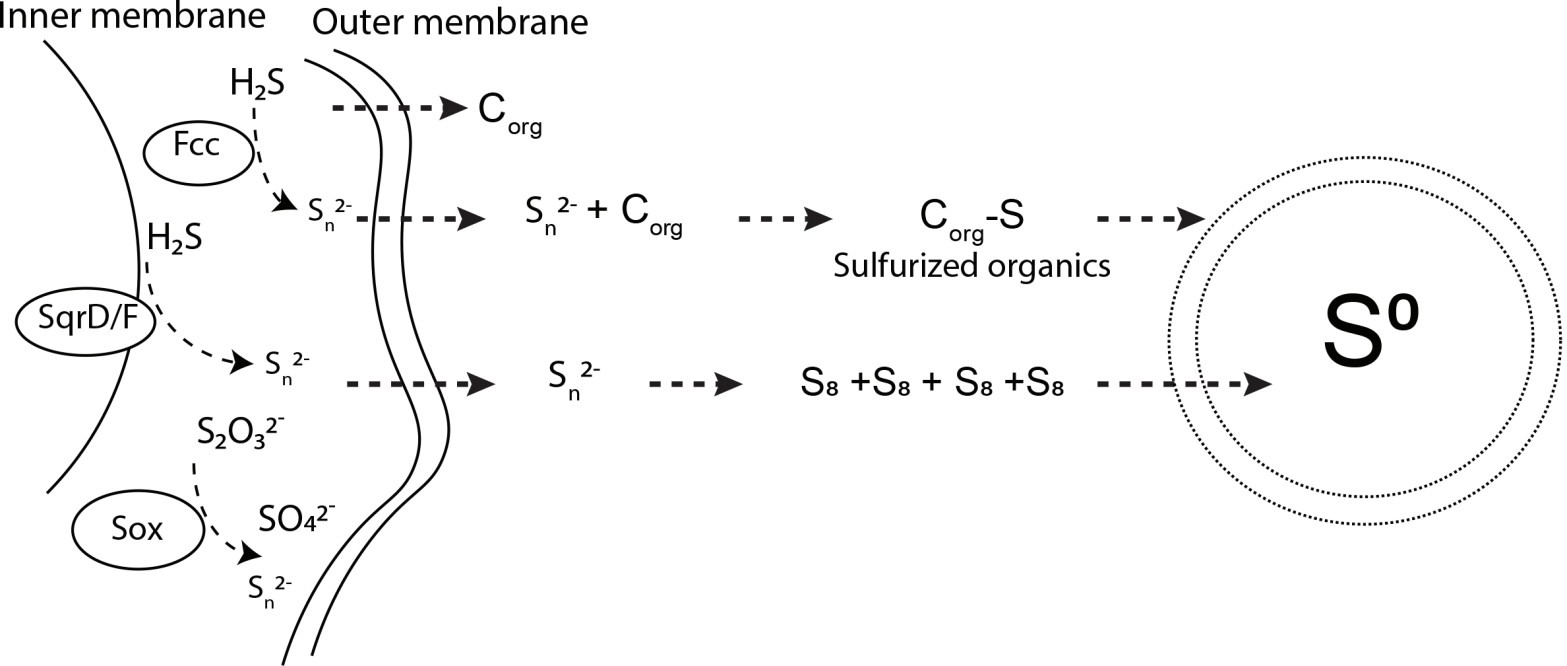
Pathways for sulfur oxidation and extracellular S(0) precipitation by *Sulfuricurvum kujiense. SqrD/F* and *fcc* genes are used to oxidize sulfide and produce polysulfides (Frigaard and Bryant, 2008; Handley et al., 2014). The *SoxABXYZ* pathway produces polysulfides (S_n_^2−^) or sulfate (SO_4_^2−^) (Frigaard and Bryant, 2008). Polysulfides produced in the periplasm diffuse to the exterior of the cell wall, where S_n_^2−^ undergo concatenation to form S_8_ rings. These S_8_ rings then precipitate as solid S(0) particles with different crystal structures. Dissolved organics excreted by *S. kujiense* undergo sulfurization upon reaction with polysulfides. Sulfurized organics self-assemble to produce the organic envelope of the S(0) globules and other particulate S(0) morphologies, which is depicted by the double membrane layer encapsulating S(0). See the “Discussion” text for further details on this mechanism.

Based on our experimental results, and our knowledge of the S(0) organomineralization mechanism described in Cosmidis et al. (2019), we can propose a model for the extracellular formation of S(0) by *S. kujiense* (Figure 7). *S. kujiense* oxidizes reduced sulfur compounds (e.g., sulfide, thiosulfate) using enzymes located in the inner membrane (e.g., *SqrD* and *SqrF*), as well as *Fcc,* and the *SoxXYZAB* pathway, located in the periplasm (based on Candidatus *Sulfuricurvum* sp. RIFRC-1; Handley et al., 2014). Precipitation of particular S(0) outside of the cells thus requires the involvement of a soluble sulfur intermediate that can diffuse or be exported through the outer membrane (Frigaard and Bryant, 2008). Experimental results from the study of another sulfur oxidizer precipitating S(0) extracellularly, *C. tepidum*, suggested that this intermediate could be polysulfides (Marnocha et al., 2016). Our own results are compatible with the presence of long chain inorganic polysulfides, as well as organic di- or polysulfides, based on the presence of a tall peak at 2472.0 eV in the S K-edge XANES spectrum of the solution from a 4-day-old *S. kujiense* culture (Figure 6B; Supplementary Table S3). We note that this peak is absent from the spectrum acquired on the solution from a 6 months-old *S. kujiense* spent medium (Figure 6D; Supplementary Table S3), showing that with time this intermediate will fully oxidize and/or precipitate out of solution with S(0). Inorganic polysulfides are known to cyclize to form S_8_ rings (Steudel and Eckert, 2003; Garcia and Druschel, 2014), leading them to eventually crystallize as cyclooactasulfur allotropes outside of the cells. In the case of organic di- or polysulfides, it is not clear how these intermediates would interact to induce S(0) precipitation.

Inorganic polysulfides are strong nucleophiles and, hence, strongly reactive with respect to organic molecules (Krein and Aizenshtat, 1994; Amrani and Aizenshtat, 2004; Amrani et al., 2007). As previously shown by others, the reaction of organics with polysulfides leads to the rapid sulfurization of these organic molecules, and results in the formation of low and high molecular weight organosulfur compounds, that can be cross-linked by mono- or polysulfide bonds (Schouten et al., 1994; Kok et al., 2000; van Dongen et al., 2003; Pohlabeln et al., 2017). It has been proposed that these sulfurization reactions could eventually result in the formation of amphiphilic molecules that self-assemble into organic envelopes or vesicles surrounding the organomineralized S(0) particles (Cosmidis et al., 2019). Since S_8_ is hydrophobic, it tends to form and remain within these vesicles, stabilized at the contact of the hydrophobic groups of the organic molecules and protected from the aqueous environment. As described in previous studies (Lau et al., 2017; Cosmidis et al., 2019), the close interaction between the S_8_ minerals and their organic envelopes could furthermore explain the formation and persistence of metastable allotropes (β- and γ-S_8_) in the cultures and spent media of *S. kujiense*.

We therefore propose that *S. kujiense* use extracellular soluble organics to form and stabilize S(0) extracellularly (Figure 7). We note that this model could also explain observations made on the extracellular S(0) globules produced by *C. tepidum*, such as the fact that they can form at distance from the cells (Marnocha et al., 2016), or that they are coated by a layer of organics (Marnocha et al., 2019).

Whether or not other SOB biomineralizing S(0) extracellularly use a similar mechanism is an exciting question for future research. To answer it, a better knowledge of the properties of extracellular S(0) particles formed by a wider range of bacteria and archaea is needed. Surprisingly, the vast majority of studies focusing on determining the chemical form and structure of microbial sulfur have focused on intracellular S(0) globules (e.g., Pasteris et al., 2001; Pickering et al., 2001; George et al., 2008; Berg et al., 2014).

The extracellular S(0) globules produced by *Acidithiobacillus ferrooxidans* have been characterized by high performance liquid chromatography and XANES spectroscopy (Steudel et al., 1987; Prange et al., 2002) and were determined to be composed mostly of polythionates. However, polythionates are only stable at low pH, so this model probably does not apply to microorganisms precipitating S(0) at near-neutral pHs (Kleinjan et al., 2003). Extracellular S(0) produced by the phototrophic green sulfur bacterium *Chlorobium vibrioforme* was found to be composed mostly of sulfur chains terminated by organic residues (Prange et al., 2002). More work is needed to evaluate the diversity of extracellular microbial S(0) forms, and to determine whether or not organics are involved in their formation and stabilization.

It is more than likely that organics are also involved in the formation and stabilization of intracellular S(0) globules by bacteria, although it is not known whether a mechanism similar to that described in the present study is involved. Interestingly, intracellularly biomineralized S(0) often possesses characteristic properties of organomineralized S(0), such as spherical morphologies (Dahl and Prange, 2006), metastable structures (e.g., amorphous S_8_; Nims et al., 2019), or the presence of an organic coating (Strohl et al., 1981; Prange et al., 2004).

### The Biological Function of S(0) Organomineralization

The results of our experiments demonstrate that extracellular organics produced by *S. kujiense* are necessary to form stable S(0) particles outside of the cells. It is not clear at this point whether the organics involved in this process are being produced specifically for this purpose, or if they are waste products or extracellularly secreted metabolites with other biological functions. Another important question that remains to be addressed is whether extracellular S(0) production presents any advantage compared with intracellular S(0) biomineralization, other than the fact that larger amounts of S(0) can be stored without the physical constrain of the microbial cell wall. Regardless, organomineralizing sulfur extracellularly could be a strategy used by the cells to store S(0) that can be further oxidized or reduced to gain energy when environmental conditions fluctuate, as they commonly do in the redox gradient habitats of sulfur oxidizing neutrophiles (Hamilton et al., 2015). Indeed, *S. kujense* is able to use S(0) as an electron donor for growth (Kodama and Watanabe, 2003). It is possible that the special properties of organomineralized S(0), and in particular its organic envelope and metastable crystal structure, make it a particularly bioavailable form of S(0). Previous experimental studies have highlighted several factors that might control the bioavailability of extracellular S(0), such as crystal structure and surface area (Laishley et al., 1986; Marnocha et al., 2019), chemical form (S_8_ rings versus S_μ_ chains; Franz et al., 2007), and presence of an organic coating (removing the hydrophobic barrier that prevents the cells from interacting with sulfur; Knickerbocker et al., 2000; Marnocha et al., 2019). For instance, *C. tepidum* is able to grow from the oxidation of its own S(0) globules, but is not capable of growth on commercial S(0) (Marnocha et al., 2019). Using organomineralization processes, ecologically relevant properties of S(0) particles can now be finely controlled in the laboratory (Cosmidis et al., 2019). This opens the way for future studies that will test the influence of these different factors (especially, crystal structure and presence of an organic coating) on S(0) utilization by S-oxidizing, S-reducing, or S-disproportionating microorganisms.

## Conclusion

This study provides the first experimental evidence of the importance of organomineralization in extracellular S(0) formation by sulfur-oxidizing microorganisms. The presence of microbial organics not only determine whether or not stable particulate S(0) is produced, but also controls the properties (morphology, organic coating, and crystal structure) of the extracellular S(0) minerals. If this organomineralization model is applicable to other sulfur-oxidizing microorganisms, it could be of great importance for controlling the stability, reactivity, and possibly bioavailability, of S(0) in natural environments, and thus play a very central role in the microbial sulfur cycle.

## Data Availability Statement

The raw data supporting the conclusions of this manuscript will be made available by the authors, without undue reservation, to any qualified researcher.

## Author Contributions

BC, PH, CC, JM, and JC designed the study. BC performed the experiments, and collected and processed the analytical data under the guidance of JM and JC. BC, JM, and JC contributed to the scientific interpretation of the data. BC prepared the figures, and BC and JC wrote the manuscript text. All authors reviewed, contributed to, and approved the final manuscript.

### Conflict of Interest

The authors declare that the research was conducted in the absence of any commercial or financial relationships that could be construed as a potential conflict of interest.

## Footnotes

^1^unicorn.mcmaster.ca/aXis2000.html
