# Supplementary material for: Elemental Sulfur Formation by Sulfuricurvum kujiense Is Mediated by Extracellular Organic Compounds
Source: Front Microbiol. 2019 Nov 27;10:2710. doi: 10.3389/fmicb.2019.02710 (PMC6890823; doi:10.3389/fmicb.2019.02710)
Supplement: Supplementary file 1 [file Presentation_1.pdf]

Supplementary Material

1 Supplementary Figures

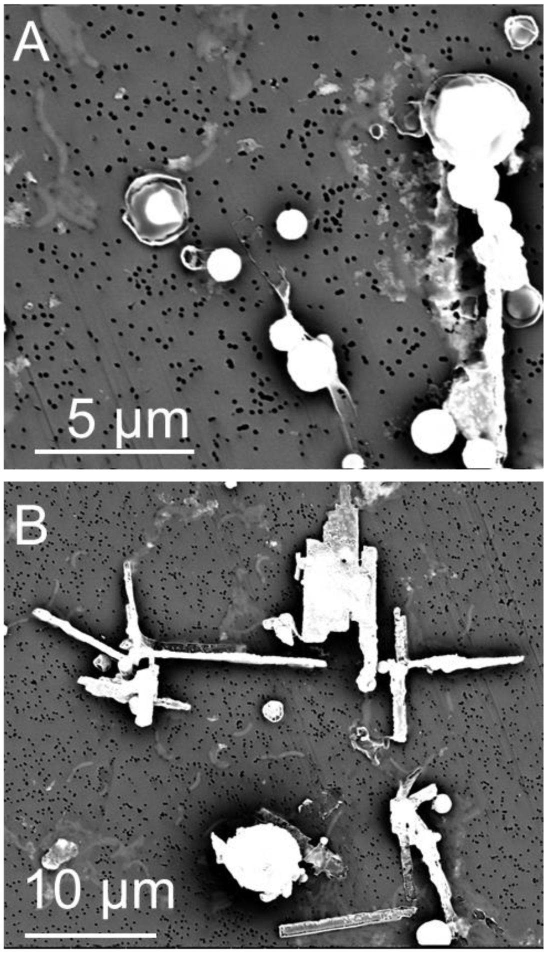

Supplementary figure 1. SEM images of particles formed in *S. kujiense* cultures with thiosulfate and sulfide after 4 days of growth. The images were acquired in the in backscattered electron mode (bright areas are sulfur-rich).

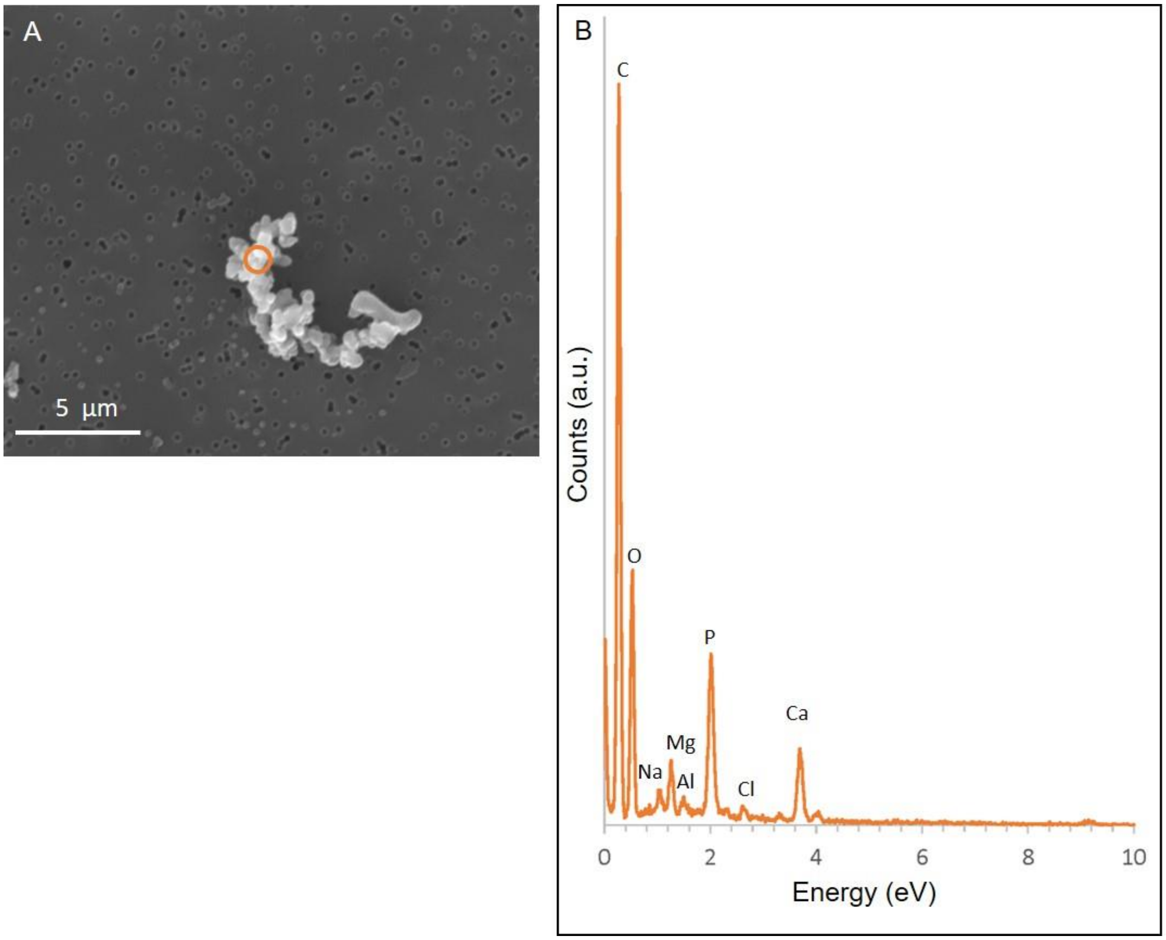

Supplementary figure 2. Phosphate particles found in *S. kujiense* cultures with thiosulfate only after 5 weeks of growth. (**A**) SEM micrograph. (**B**) XEDS analysis collected on the area depicted by an orange circle in (A).

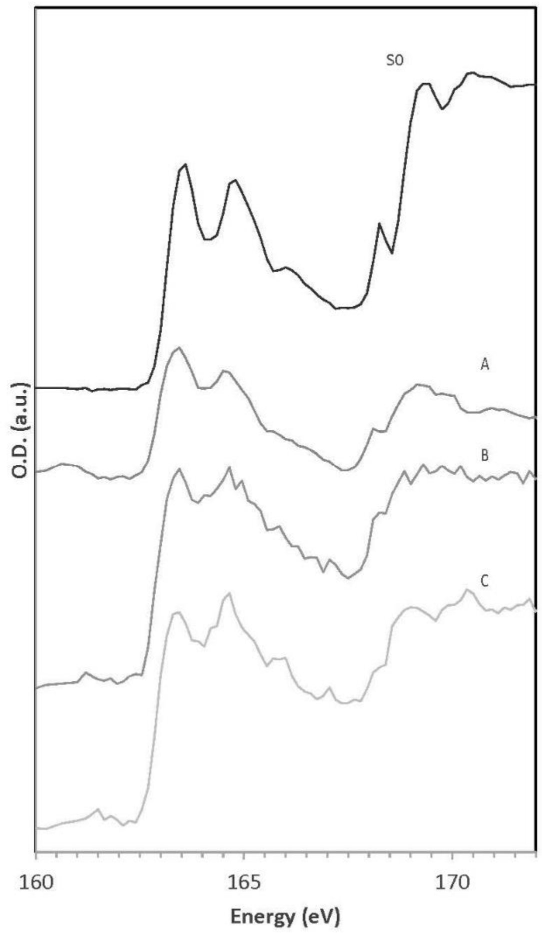

Supplementary figure 3: S L-edge STXM/XANES analyses obtained on the S(0) particles found in *S. kujiense* cultures with thiosulfate and sulfide and shown in Fig. 3. Spectrum A corresponds to the area in the dashed box in Fig. 3D, spectrum B to area in the dashed box in Fig. 3E, and spectrum C to the area in the dashed box in Fig. 3F. A reference elemental sulfur spectrum (S0) is also shown.

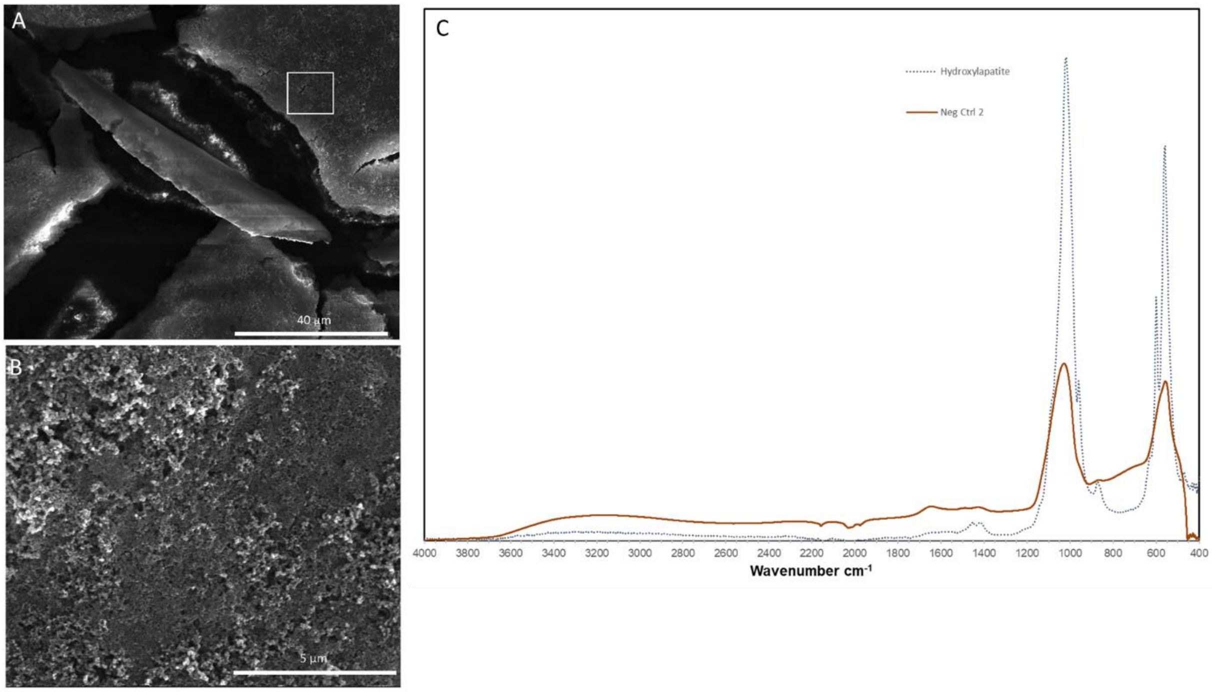

Supplementary figure 4: Phosphate particles precipitated in the abiotic control of the “spent medium” experiment. (A,B) SEM images. (B) is a close-up on the area depicted by a white box in (A). (C) FTIR spectrum collected on these particles (brown line), compared with a hydroxyapatite reference spectrum (broken grey line) (RRUFF database).

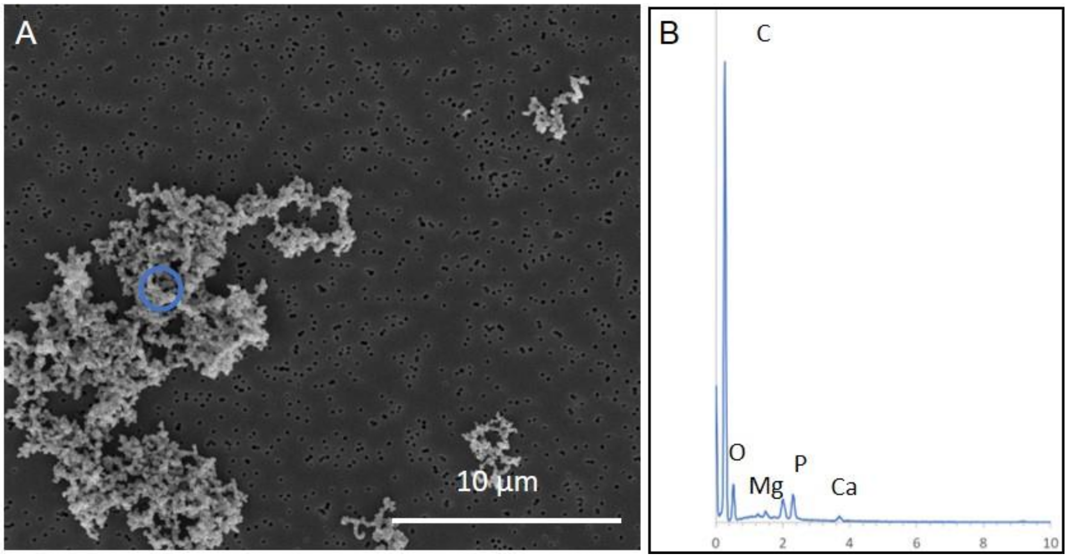

Supplementary figure 5. Phosphate particles found in the spent medium experiment prepared with *S. kujiense* cell cultures in the exponential phase. (A) SEM micrograph. (B) XEDS analysis collected on the area depicted by a blue circle in (A).

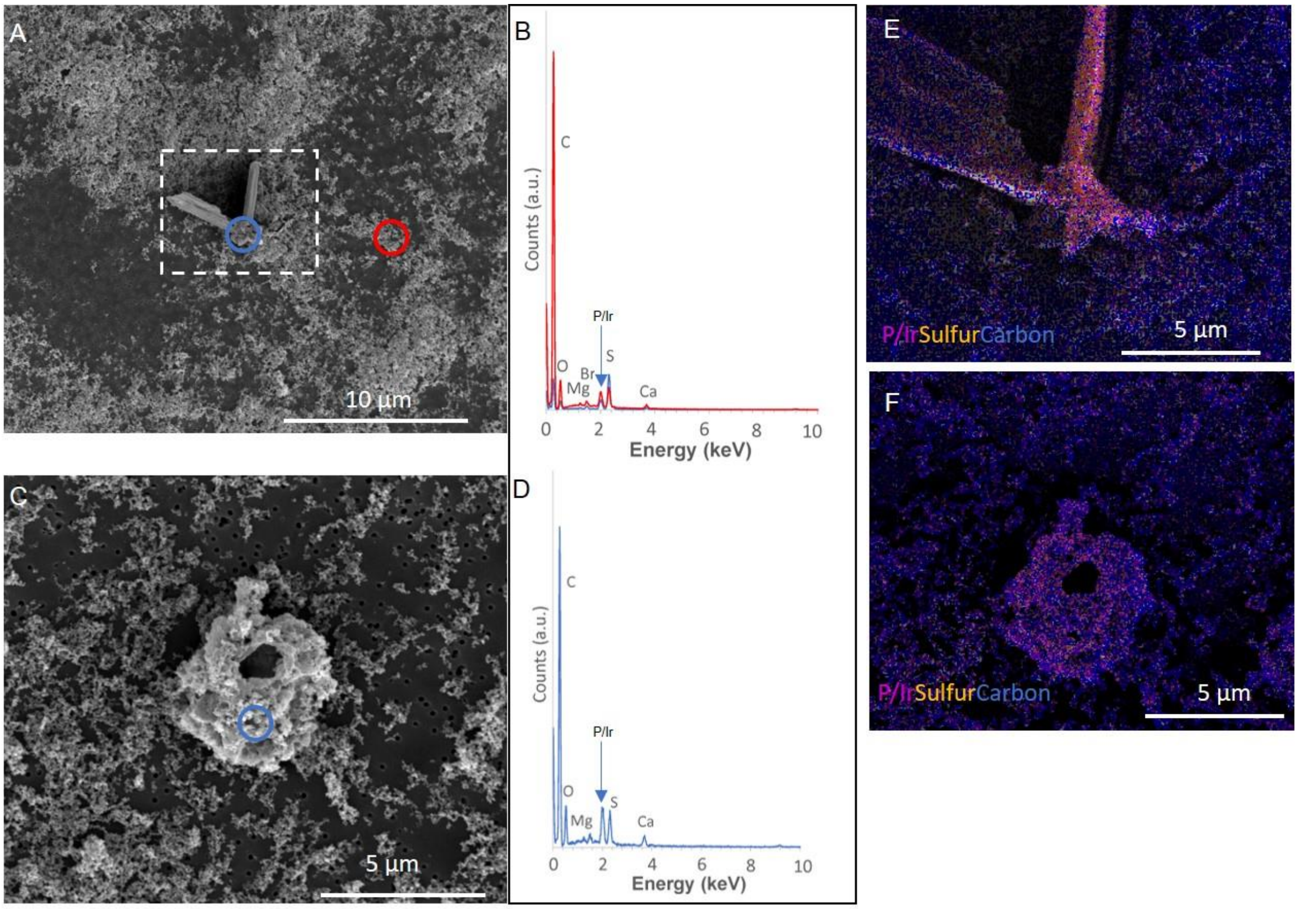

Supplementary figure 6: Phosphate and sulfur particles precipitated in the *E. coli* spent medium experiment after 1 month. (**A,C**) SEM images (**B,D**) XEDS spectra obtained on the areas depicted by circles in (A) and (C); (**E**) is an XEDS map of a subset of (A), the map shows the distribution of phosphorous, sulfur, and carbon. Note that the XEDS peak for Ir (coating) at ~2 keV interferes with that of P. (**F**) is an XEDS map showing the distribution of phosphorous, sulfur, and carbon in the area shown in (C).

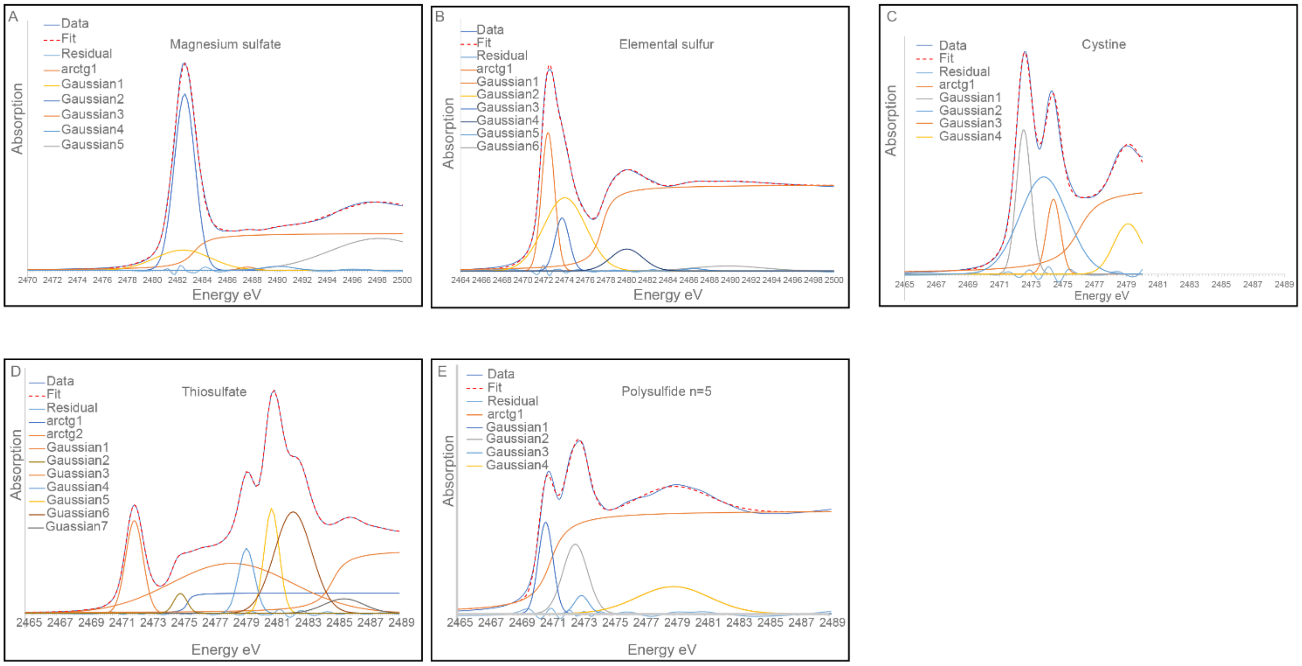

Supplementary figure 7. Gaussian curve fitting of S K-edge XANES spectra of the following standards: (A) magnesium sulfate; (B) elemental sulfur; (C) cystine; (D) sodium thiosulfate; (E) polysulfides (S_n^{2-}, n=5).

2 Supplementary Tables

Supplementary Table 1. Summary of conditions and sampling times for *S. kujiense* culture experiments. Sodium sulfide (Na_2S) and thiosulfate ($Na_2S_2O_3$) are indicated as the electron donors.

**Electron donor**	**Sampling time**	**Analyses**
2mM Na_2S + 10 mM $Na_2S_2O_3$	4 days	SEM
2 mM Na_2S + 10 mM $Na_2S_2O_3$	2 weeks	Raman, SEM
2 mM Na_2S + 10 mM $Na_2S_2O_3$	5 weeks	STXM
10 mM $Na_2S_2O_3$	11 days	SEM
10 mM $Na_2S_2O_3$	4 days	S K-edge XANES

Supplementary Table 2. Summary of conditions and sampling times for *S. kujiense* spent medium experiments

**Growth phase of the initial culture**	**Sampling time**	**Analyses**
Exponential	5 weeks	SEM
Stationary	5 weeks	FT-IR
Stationary	6 weeks	Correlative Raman-SEM
Stationary	6 months	S K-edge XANES
Abiotic control	6 months	SEM, S K-edge XANES

Supplementary Table 3. Gaussian curve fitting decomposition of the S K-edge XANES spectra from the spent medium experiments (Figure 6) and from the standards (Figure S7).

	Energy	Area	Width	χ^2	r-factor
Sulfuricurvum kujiense spent medium experiment					
Arctg1	2484		3.25	0.38	0.0015
Gauss1	2481.3	4.24			
Gauss2	2482.75	2.87			
Gauss3	2487	0.36			
Escherichia coli spent medium experiment					
Arctg1	2474		0. 72	0.40	0.0015
Arctg2	2484		0.26		
Gauss1	2472.2	1.24			
Gauss2	2481.3	5.4			
Gauss3	2482.75	1.9			
DSMZ media 1020 with thiosulfate (blank)					
Arctg1	2474		0.07	0.19	0.0007
Arctg2	2484		0.7		
Gauss1	2472	0.38			
Gauss2	2473.59	0.23			
Gauss3	2476.3	0.21			
Gauss4	2479.92	3.3			
Gauss5	2481.3	1.5			
Gauss6	2482.75	3.3			
Gauss7	2488.34	0.09			
Sulfuricurvum kujiense culture					
Arctg1	2472.59	0.6		0.42	0.0014
Arctg2	2484	2.23			
Gauss1	2472	1.37			
Gauss2	2479	1.1			
Gauss3	2480.9	0.7			
Gauss4	2482.4	2.44			

	Energy	Height	Width	χ^2	r-factor
Elemental sulfur					
Arctg1	2477.64	1	0.74	0.06	0.00025
Gauss1	2472.45	2.54	0.63		
Gauss2	2473.8	1.05	0.69		
Gauss3	2474.07	4.34	2.05		
Gauss4	2480.04	0.98	1.54		
Gauss5	2486.54	0.06	1.01		
Gauss6	2489.81	0.48	3.37		
Thiosulfate ($Na_2S_2O_3$)					
Arctg1	2475.41	0.275	0.22	0.04	0.00018
Arctg2	2484.71	0.863	0.7		
Gauss1	2472.01	1.75	0.56		
Gauss2	2474.97	0.29	0.43		
Gauss3	2478.27	6.54	3.9		
Gauss4	2479.21	1.12	0.51		
Gauss5	2480.82	1.68	0.48		
Gauss6	2482.2	4.09	1.19		
Gauss7	2485.46	0.63	0.28		
Cystine					
Arctg1	2476	1	1	0.14	0.00068
Gauss1	2472.52	1.9	0.46		
Gauss2	2473.78	4.16	1.51		
Gauss3	2474.4	0.93	0.43		
Gauss4	2479.11	1.30	0.9		
Polysulfides (S_n^{2-}, n = 5)					
Arctg1	2471		0.8	0.09	0.0004
Gauss1	2470.7		1.04		
Gausss2	2472.6		1.33		
Gauss3	2473		0.2		
Gauss4	2478.9		1.51		

	Energy	Height	Width	χ^2	r-factor
Sulfate ($MgSO_4$)					
Arctg1	2483.32	1	0.87	0.12	0.00033
Gauss1	2482.44	3.03	2.23		
Gauss2	2482.57	9.96	0.84		
Gauss3	2487.6	0.14	0.64		
Gauss4	2490.08	0.33	1.22		
Gauss5	2498.15	8.51	3.97		
